# Screening mental health risks for adolescents in South Korea

**DOI:** 10.3389/fpsyg.2025.1589136

**Published:** 2025-07-16

**Authors:** Euntaek Hong, Joohee Lee, Seowon Yoon, Surin Cho, Yong-Chun Bahk, Jeongwon Choi, Kee-Hong Choi

**Affiliations:** ^1^School of Psychology, Korea University, Seoul, Republic of Korea; ^2^Department of Psychology, Michigan State University, East Lansing, MI, United States; ^3^Samsung Medical Center, Seoul, Republic of Korea; ^4^Korea University Mental Health Institute, Sejong, Republic of Korea; ^5^National Youth Policy Institute, Sejong, Republic of Korea

**Keywords:** suicide risk, depression, anxiety, adolescence, validation, screening tool, public mental health, COVID-19

## Abstract

**Background:**

Depression, anxiety, and suicidal ideation are significant mental health problems among adolescents, especially in South Korea, which has one of the highest adolescent suicide rates globally. However, few standardized and validated mental health screening tools exist for this population. This study aimed to examine the psychometric properties and establish normative data for the Mental Health Screening Tool for Depressive Disorder (MHS: D), Generalized Anxiety Disorder (MHS: A), and Suicide Risk (MHS: S) in South Korean adolescents.

**Methods:**

An online survey involving 6,689 students and out-of-school youths (aged 10–18 years) was conducted between July and August 2021. Psychometric properties—including reliability, validity, and measurement invariance—were assessed by comparing adolescent and adult samples and demographic subgroups within the adolescent sample.

**Results:**

All three screening tools demonstrated excellent internal consistency (Cronbach’s α: MHS: D = 0.92, MHS: A = 0.93, and MHS: S = 0.86) and adequate unidimensional factor structure in the adolescent sample. A multi-group confirmatory factor analysis showed that the unidimensional factor structure of each instrument was maintained between the adolescent and adult samples, and that measurement invariance was maintained across adolescent sex and age subgroups. Reference norms indicated higher symptom prevalence among girls compared to boys, with symptom severity increasing with age. Significant correlations with mental health indicators (i.e., somatization, self-harm, perceived stress, and peritraumatic COVID-19 stress) supported the high construct validity of the instruments and highlighted the detrimental impact of mental health concerns on overall well-being.

**Conclusion:**

The MHS: D, MHS: A, and MHS: S demonstrated excellent psychometric properties across sex and age subgroups in a representative adolescent sample. Using these validated tools in clinical and community settings can aid in monitoring adolescent mental health and preventing suicide risk.

## 1 Introduction

### 1.1 Background

Approximately 10–20% of adolescents worldwide experience psychological problems (Barican et al., 2022; Kieling et al., 2011). A significant proportion of mental health issues first appear during adolescence (Merikangas et al., 2010). Conditions such as depression and anxiety during this critical period can lead to functional impairment (Dunn and Goodyer, 2006) and chronic mental illness (Kessler and Wang, 2008), resulting in increased suicide risk (Balazs et al., 2013). South Korea’s highly competitive educational environment contributes to a high prevalence of depression and anxiety among the youth, leading to increased suicide risk and the world’s highest youth suicide rate for over 20 years (Jung and Cho, 2020; Kim, 2018). South Korea’s youth suicide rate reached a record high of 11.7 deaths per 100,000 in 2021 (Ministry of Gender Equality and Family, 2023). Immediate preventive measures and interventions are critical to reducing the adolescent suicide rate in South Korea, highlighting the importance of accessible and effective screening for youth depression, anxiety, and suicide at an early stage. To accurately detect mental health concerns among adolescents, screening instruments must be validated to ensure they are suitable for assessing psychological problems in this demographic (Mangione et al., 2022).

For accurate mental health screening of South Korean adolescents, screening instruments must demonstrate strong psychometric properties—such as internal consistency and validity—and consistently assess adolescents’ mental health across different sex and age groups. Mental health fluctuates by age and sex during adolescence, with the prevalence rates of depression, anxiety, and suicide risk being lower in early adolescence and tending to increase during mid-adolescence (Copeland et al., 2014; Costello et al., 2003; Nock et al., 2013) and being higher in female adolescents compared to their male counterparts (Lu, 2019; Merikangas et al., 2010; Nock et al., 2013; Rice et al., 2019). It is also important to keep in mind that adolescent developmental differences in the phenomenology of depression and anxiety (Weiss and Garber, 2003; Zahn–Waxler et al., 2000), as well as the prevalence, could have significant impact on mental health screening.

Therefore, widely used MHS instruments such as the Patient Health Questionnaire-9 (PHQ-9) (Kroenke et al., 2001), 7-item Generalized Anxiety Disorder Scale (GAD-7) (Spitzer et al., 2006), and Suicidal Behaviors Questionnaire (SBQ) (Osman et al., 2001) have been validated for use with the adolescent population, including measurement invariance analysis (Adjorlolo et al., 2022; Kliem et al., 2024; Löwe et al., 2008a; Richardson et al., 2010; Romano et al., 2022; Tiirikainen et al., 2019). However, measurement consistency of these tools across demographic subgroups has not yet been established for South Korean adolescents. Also, to our knowledge, there are no studies of depression, anxiety, and suicide risk screening scales for adolescents that include normative data, although some exist for South Korean adults (Kang et al., 2021; Shin et al., 2020). Since scores of mental health screeners typically do not follow a normal distribution, only presenting T- or Z-scores without normative percentiles is insufficient (Crawford et al., 2009). Before using screening tools for adolescents, their psychometric properties and measurement consistency must be evaluated, and normative reference data with percentile ranks should be established in a representative sample.

### 1.2 MHS tool for South Korean adolescents

In South Korea, mental health screening tools (MHS) were developed to facilitate the early detection and prevention of mental health disorders, targeting depressive disorder (MHS: D) (Park et al., 2022), generalized anxiety disorder (MHS: A) (Kim et al., 2021), and suicide risk (MHS: S) (Yoon et al., 2020). These tools were systematically created over a 3-year research period through literature reviews, expert consultations, and preliminary studies with both clinical and non-clinical participants to ensure item validity and refinement. Each tool underwent evaluation for convergent and discriminant validity through comparisons with existing psychological measures. The MHS: D was evaluated against the BDI-II (Beck et al., 1996; Park et al., 2020), PHQ-9 (Cho and Kim, 1998; Kroenke et al., 2001), and CES-D (Cho and Kim, 1998; Radloff, 1977); the MHS: A was assessed through comparisons with the GAD-7 (Ahn et al., 2019; Spitzer et al., 2006), BAI (Beck et al., 1988; Oh et al., 2018), and PSWQ (Lim et al., 2008; Meyer et al., 1990). The MHS: S established optimal items and cutoff points based on suicide risk criteria. The Mini-International Neuropsychiatric Interview (Sheehan et al., 1998; Yoo et al., 2006) served as the diagnostic reference standard, with blinded procedures implemented to minimize bias and ensure reliability.

Each tool demonstrated higher diagnostic sensitivity and specificity than existing measures (Kim et al., 2021; Park et al., 2022; Yoon et al., 2020). The MHS: D effectively identified depressive disorders, while the MHS: A proved capable of detecting generalized anxiety disorder at an early stage. The MHS: S exhibited strong accuracy in assessing suicide risk across different severity levels. They all demonstrated appropriate psychometric properties, including internal consistency and convergent validity, and can be effectively applied in clinical settings. Additionally, their concise and efficient format makes them highly adaptable for use in online mental health services and applications.

While MHS tools can adequately measure depressive and anxiety symptoms and suicide risk in South Korean adult samples, the psychometric properties and normative data of these tools have not yet been validated among adolescents. Further research is, therefore, needed to establish the validity, reliability, measurement invariance, and normative data of MHS tools in Korean youth sample, considering adolescents’ developmental characteristics.

### 1.3 Study aims

This study examined the psychometric properties of the MHS: D, MHS: A, and MHS: S and provided the normative data of these tools in a nationally representative sample of South Korean adolescents aged 10–18 years. We first examined the internal consistency, factor structure, and validity of each tool within an adolescent cohort. Subsequently, multi-group confirmatory factor analysis (MGCFA) was performed to verify the measurement consistency between adult and adolescent samples and between different sex and age (school grade) subgroups within the adolescent cohort. We examined various aspects of the mental health status of South Korean adolescents to investigate the construct validity of MHS tools and the impact of common mental health concerns on other clinical symptoms.

The specific aims of this study were to

1. Examine the internal consistency, item response characteristics, and factor structure of each screening tool within the adolescent population.
2. Conduct MGCFA to ascertain the measurement invariance of each tool between the adolescent and adult samples, and between age (school grade) and sex subgroups within the adolescent population.
3. Generate normative data according to sex and different age categories.
4. Examine correlations between screening tools and other mental health aspects.

## 2 Materials and methods

### 2.1 Participants

This study used data from the “National Survey on the Mental Health Status of Teenagers,” conducted by the National Youth Policy Institute in 2021. This survey assessed the prevalence of mental health problems among out-of-school adolescents (those who had dropped out of, or never enrolled in school), their exposure to mental health protective and risk factors during the COVID-19 pandemic, and their awareness and use of community mental health services. Participants were selected using stratified cluster sampling, resulting in a nationwide sample of 5,937 students from 4th grade in elementary school to 3rd grade in high school. Additionally, 752 out-of-school adolescents aged 10–18 years were randomly sampled from 220 regional dream centers across the country. Data were collected from July to August 2021, during the social distancing period in South Korea, through a self-administered online survey.

To verify the measurement invariance of the MHS tools between the adolescent and adult groups, data from the online versions of the MHS: D, MHS: A, and MHS: S that were collected in an adult group validation study were employed (Kim et al., 2021; Park et al., 2022; Yoon et al., 2020). This dataset included 527 adults, with 270 participants consecutively sampled from those visiting university hospitals and the remainder recruited via online advertisements. The mean age of the adult participants was 38.6 years, with 340 female participants (64.5%).

### 2.2 Instruments

General demographic characteristics (e.g., sex, age, school, cigarette and alcohol use, sleep quality) and clinical scales pertaining to MHS tools were selected from the “Survey on the Mental Health Status of Teenagers” (Choi et al., 2021), as detailed below. To measure the correlation between MHS tools and negative mental health outcomes, this study employed self-report scales for somatization, self-harm behavior, perceived stress, and COVID-19 peritraumatic distress.

#### 2.2.1 MHS tool for depression

The MHS: D is a self-report measure for the screening and intervention for major depressive disorder in mental health service settings (Park et al., 2022). It comprises 12 items enquiring about symptoms of major depressive disorder over the preceding 2 weeks, with responses scored on a five-point Likert scale from 0 (*not at all*) to 4 (*extremely*). The overall score reflects symptom severity. Higher scores for items 10 and 11, which measure significant changes in appetite, contribute to the total score. The MHS: D demonstrated excellent validity and reliability in a validation study using a South Korean adult sample, including a clinical population. A total score of 8–11 indicates mild symptoms, 12–19 indicates moderate symptoms, and 20 or higher indicates severe symptoms. A cutoff score of 17 indicates major depressive disorder. The internal consistency of the MHS: D was excellent (Cronbach’s alpha: 0.95).

##### 2.2.2 MHS tool for anxiety

The MHS: A is a self-report measure used for screening and intervention for generalized anxiety disorder in mental health service settings (Kim et al., 2021). It comprises 11 items enquiring about symptoms of generalized anxiety disorder over the preceding 2 weeks, with responses scored on a five-point Likert scale from 0 (*not at all*) to 4 (*extremely*). The overall score reflects symptom severity. The MHS: A demonstrated excellent validity and reliability in a validation study with a South Korean adult sample, including a clinical population. A total score of 10–19 indicates mild anxiety, 20–29 indicates moderate anxiety, and 30 or higher indicates severe anxiety. A cutoff score of 15 indicates generalized anxiety disorder. The MHS: A showed excellent internal consistency (Cronbach’s alpha: 0.97).

##### 2.2.3 MHS tool for suicide risk

The MHS: S is a self-report measure for screening and intervention for suicide risk in mental health service settings (Yoon et al., 2020). It comprises four items enquiring about suicide-related symptoms over the preceding 2 weeks, with responses scored on a five-point Likert scale from 0 (*never*) to 4 (*always*). The overall score reflects symptom severity, considering a score of 1–2 as positive risk, and 3 or higher as high risk. The MHS: S demonstrated excellent validity and reliability in a validation study with a South Korean adult sample, including a clinical population. The internal consistency of the MHS: S online test in the adult sample was good (Cronbach’s alpha: 0.82).

##### 2.2.4 Korean children’s somatization inventory

The K-CSI is a self-reported measure of somatic symptoms adapted from Walker et al.’s original inventory (Walker et al., 1991). It assesses the distress caused by 36 physical symptoms over the preceding week, with responses scored on a four-point Likert scale from 0 (no symptoms) to 3 (very severe). The K-CSI demonstrated good validity in a study on Korean adolescents (Cronbach’s alpha: 0.87) (Shin, 2003). In this study, the internal consistency of the scale was good (Cronbach’s alpha: 0.87).

##### 2.2.5 Korean version of the self-harm inventory

The Korean version of the Self-Harm Inventory (K-SHI) is a self-report measure of intentional self-harm behavior adapted from the original scale by Sansone et al. (1998). It comprises 22 items enquiring about self-harm behavior within the last 6 months, with responses scored as either 1 (yes) or 0 (no). Following a validation study with Korean adolescents (Kim et al., 2019), the Institutional Review Board of the Korea Youth Policy Institute recommended modifications to the scale to protect respondents’ rights because of the sensitive nature of certain items (Choi et al., 2011). Consequently, only five items were selected to assess self-harm behavior. In this study, the internal consistency of the K-SHI was good (Cronbach’s alpha: 0.88).

##### 2.2.6 Korean perceived stress scale for adolescents

The Korean perceived stress scale for adolescents (KPSS-A) is a self-report measure of perceived stress adapted from the original scale (Cohen et al., 1983). It comprises 10 items, with responses scored on a five-point Likert scale from 0 (*never*) to 4 (*very often*). The KPSS-A has been validated in a cohort of South Korean adolescents (Cronbach’s alpha: 0.90) (Yoon and Kim, 2019), and its internal consistency in this study was adequate (Cronbach’s alpha: 0.72).

##### 2.2.7 COVID-19 peritraumatic distress index

The COVID-19 peritraumatic distress index (CPDI) is a self-report measure for traumatic distress related to COVID-19, encompassing symptoms of depression, anxiety, specific phobias, cognitive changes, avoidance and compulsive behavior, physical symptoms, and reduced social functioning (Qiu et al., 2020). It comprises 24 items, with responses scored on a five-point Likert scale from 0 (*not experienced at all*) to 4 (*almost always experienced*). Higher scores indicate greater distress. For the survey, the Korean version was developed through cross-translation by two bilingual individuals, and the reverse translation was subsequently validated (Choi et al., 2021). The internal consistency of the CPDI in this study was excellent (Cronbach’s alpha: 0.91).

### 2.3 Statistical analysis

The mean scores, item intercorrelations, and internal consistency of each tool (MHS: D, MHS: A, and MHS: S) were calculated to assess the psychometric characteristics of the screening tools. To test the internal consistency of the tools, Cronbach’s α of total items and coefficients when individual items were deleted were measured. CFA was conducted to confirm the factor structure of each tool.

To ensure that the consistency of the measurement was maintained across adult and adolescent populations, as well as across sex and age groups (elementary school: aged 11–13 years, middle school: aged 14–16 years, high school: aged 17–19 years), we tested for measurement invariance using MGCFA. Measurement invariance refers to whether the same factor structure is maintained across different populations, such as sex, race, and age. Testing the measurement invariance of a screening tool is important to ensure that data from different groups are interpreted consistently. This study follows hierarchical successive steps according to Byrne (2011). First, a configural invariance test is conducted to verify that the factor structure is consistent across the subgroups. Configural invariance tests focus on ensuring that the same constructs exist within each group and that each construct is measured by the same observed variable. In this phase, differences in factor loadings and intercepts are allowed. Second, if configural invariance holds, a metric invariance test is conducted to ensure that the item means are equivalent across subgroups, by restricting factor loadings to be equal across subgroups. When it is confirmed that the restricted model does not have a significantly worse model fit compared to the previous baseline (configural) model, metric invariance is met, and we can move on to the next phase. If metric invariance does not hold, partial metric invariance is analyzed after releasing factor loadings of some items according to the modification index (MI). Third, if full or partial metric invariance holds, a scalar invariance test is performed to ensure that the items measure the same unit across the subgroups. This involves restricting the factor loadings to be equal across subgroups. If the goodness-of-fit of this model is not significantly worse than the previous model (metric), full scalar invariance is met. If scalar invariance did not hold, partial scalar invariance was analyzed after releasing the intercepts of some items according to the MI.

During CFA and MGCFA analysis, the following criteria were used to determine goodness-of-fit: root mean square error of approximation (RMSEA) ≤ 0.08, comparative fit index (CFI) > 0.90, standardized root mean squared residual (SRMR) ≤ 0.05 (Byrne, 2011), and invariance between the model and the less restricted one was verified based on differences in RMSEA, CFI, and SRMR. Metric invariance was determined by the following criteria: ΔCFI ≤ −0.01, ΔRMSEA ≤ 0.15, ΔSRMR ≤ 0.030, indicating that each factor loading is maintained across subgroups. Scalar invariance was determined by the following criteria: ΔCFI ≤ −0.01, ΔRMSEA ≤ 0.15, ΔSRMR ≤ 0.01, indicating that each intercept of the factor loading is maintained across subgroups (Chen, 2007). If the model did not meet the criteria, we released factor loadings or intercepts of some items and assessed the partial invariance.

To provide normative data for the MHS: D, MHS: A, and MHS: S, percentile ranks by age and sex were calculated to give the total score for each instrument. To test the construct validity of the MHS tools, we examined the correlations between the MHS total scores and clinical measures related to depression, anxiety, and suicidality as well as somatization, perceived stress, and COVID-19-related stress.

Mplus Version 8 (Muthén and Muthén, 2017) was used to conduct MGCFA, and other analyses were performed using R version 4.1.2 (R Core Team, 2021).

### 2.4 Ethical considerations

Ethical approval was provided by the Institutional Review Boards of the National Youth Policy Institute (202106-HR-Unique-009), Korea University (1040548-KU-IRB-15-92-A-1 [R-A-1] [R-A-2] [R-A-2]), and Ilsan Paik Hospital (ISPAIK 2015-05-221-009). Participants’ informed written consent was obtained.

## 3 Results

### 3.1 Sample characteristics

Table 1 shows the characteristics of 6,689 adolescents (53.6% girls). They ranged in age from 10 to 18 (*M* = 14.3, SD = 2.6) years, with 30.5% in elementary school, 29.1% in middle school, 29.2% in high school, and 11.2% were out-of-school adolescents. The MHS: D displayed excellent internal consistency (Cronbach’s α: 0.92), with a mean score of 4.88 (SD = 7.29); the MHS: A had an overall mean score of 4.35 (SD = 7.23), displaying excellent internal consistency (Cronbach’s α: 0.93); and the overall mean score for the MHS: S was 0.66 (SD = 2.00), showing good internal consistency (Cronbach’s α: 0.86). Descriptive statistics for each instrument and item are presented in Table 2.

**Table 1:** Sample characteristics (*N* = 6689).

Characteristics	
	*M* (SD)
Age	14.3 (2.6)
	***N* (%)**
Gender	
Female	3,585 (53.6)
School	
Elementary school (4th to 6th grade)	2,039 (30.5)
Middle school	1,948 (29.1)
High school	1,950 (29.2)
Total school	5,937 (88.8)
Out-of-school	752 (11.2)
Substance use experience	
Alcohol*	318 (4.8)
Cigarette*	399 (5.9)
Sleep quality	
Not sufficient**	2,922 (43.7)

^*^Responded “Yes” in the question: Have you smoked cigarette/drunk alcohol in the past year? ^**^Responded “Unlikely” or “Very unlikely” in the question: Do you think the amount of sleep you have had in the last seven days is sufficient for recovery from fatigue?

**Table 2:** Means and internal validity of mental health screening tools.

Item	Mean (SD)	α if item deleted
MHS:D		
1. Depressed mood	0.462 (0.858)	0.86
2. Loss of interest	0.440 (0.836)	0.84
3. Psychomotor agitation	0.490 (0.909)	0.91
4. Fatigue	0.458 (0.909)	0.91
5. Feeling worthless	0.363 (0.846)	0.85
6. Concentration difficulty	0.421 (0.842)	0.84
7. Thoughts of suicide	0.298 (0.782)	0.78
8. Helplessness	0.264 (0.730)	0.73
9. Hopelessness	0.343 (0.827)	0.83
10/11. Appetite*	0.819 (1.128)	1.13
12. Sleep disturbance	0.522 (1.025)	1.02
Total item	4.88 (7.29)	0.92
MHS:A		
1. Excessive anxiety	0.304 (0.766)	0.92
2. Uncontrollable worry	0.421 (0.895)	0.92
3. Restlessness	0.338 (0.805)	0.92
4. Fatigue	0.396 (0.836)	0.93
5. Attention difficulty	0.3 (0.736)	0.93
6. Irritability	0.446 (0.908)	0.92
7. Muscle tension	0.481 (0.915)	0.93
8. Insomnia	0.634 (1.068)	0.93
9. Function impairment	0.197 (0.626)	0.93
10. Chest discomfort	0.421 (0.877)	0.93
11. Feeling on edge	0.411 (0.864)	0.92
Total item	4.35 (7.23)	0.93
MHS:S		
1. No will to live	0.462 (0.858)	0.858
2. Suicide thought	0.440 (0.836)	0.836
3. Suicide plan	0.490 (0.909)	0.909
4. Past attempt	0.458 (0.909)	0.909
Total item	0.66 (2.00)	0.86

The mean and standardized deviation of each item is presented, including Cronbach’s alpha coefficient excluding the item. The mean, standardized deviation, and Cronbach’s alpha coefficient of sum scores are presented below. *used the highest scores among item 10/11, indicating the increased/decreased appetite.

### 3.2 Factor analysis

A CFA was conducted to determine whether each screening tool had a single-factor structure. The internal validity of the structure was evaluated using the goodness-of-fit index, determined using the following criteria: RMSEA ≤ 0.08, CFI > 0.90, and SRMR ≤ 0.05. The goodness-of-fit for the MHS: D, MHS: A, and MHS: S all supported a unidimensional structure: MHS: D [*χ*^2^(44) = 654.334, *p* < 0.001, CFI = 0.925, SRMR = 0.040, RMSEA = 0.064 (90% CI 0.060–0.069)]; MHS: A [χ^2^(44) = 521.380, *p* < 0.001, CFI = 0.934, SRMR = 0.040, RMSEA = 0.056 (90% CI 0.052–0.061)]; and MHS: S [χ^2^(2) = 12.507, *p* < 0.001, CFI = 0.989, SRMR = 0.018, RMSEA = 0.040 (90% CI 0.021–0.061)].

### 3.3 Measurement invariance

To ensure that the factor structure of each screening instrument was maintained in the adolescent population, we conducted an MGCFA between the MHS outcomes measured in adult validation studies and the outcomes collected in this study. Validation studies conducted on adult populations identified a single-factor structure for each instrument and reported excellent validity and reliability, with CFI ranging from 0.945 to 0.985 and SRMR ranging from 0.025 to 0.04 (Kim et al., 2021; Park et al., 2022; Yoon et al., 2020). Results showed that partial scalar measurement invariance between the adolescent and adult groups was supported for the MHS: D [χ^2^(105) = 1016.468, *p* < 0.001, CFI = 0.924, SRMR = 0.040, RMSEA = 0.067 (90% CI 0.063–0.071)]; for Item 12 (sleep disturbance), scalar measurement invariance of the MHS: D between the adult and adolescent groups was not supported. After MI analysis of each item, an intercept of item 12 was freely estimated, and partial scalar measurement invariance of MHS: D between adolescents and adults was met. Full scalar measurement invariance between the adult and adolescent groups was supported for the MHS: A [χ^2^(108) = 1094.141, *p* < 0.001, CFI = 0.914, SRMR = 0.049, RMSEA = 0.069 (90% CI 0.065–0.072)]. For the MHS: S, there was a partial scalar measurement invariance between the two groups [χ^2^(16) = 16.440, *p* < 0.001, CFI = 0.979, SRMR = 0.031, RMSEA = 0.044 (90% CI 0.032–0.058)]. For Item 2 (suicidal thoughts), the scalar measurement invariance of the MHS: S across subgroups was not supported. After examining the MI of each item, an intercept of item 2 was freely estimated, and partial scalar measurement invariance of MHS: S between adolescents and adults was met. Detailed results of the MGCFA between adult and adolescent samples are presented in Supplementary Table S1.

To ensure that the factor structure of each screening instrument was maintained across the adolescent demographic subgroups, we conducted MGCFA between the MHS outcomes of the age and sex subgroups (Supplementary Table S2). For the MHS: D, partial scalar measurement invariance across age and measurement invariance across sex were supported [age: χ^2^(168) = 745.029, *p* < 0.001, CFI = 0.925, SRMR = 0.049, RMSEA = 0.056 (90% CI 0.051–0.060); sex: χ^2^(108) = 778.317, *p* < 0.001, CFI = 0.917, SRMR = 0.049, RMSEA = 0.061 (90% CI 0.057–0.065)]. Scalar measurement invariance between age subgroups was not supported for Items 10/11 (appetite change) and 3 (psychomotor agitation). Based on MI analysis of each item, intercepts of Item 10/11 (all groups), and Item 3 (elementary) were freely estimated, and partial scalar measurement invariance of MHS: D between age groups was met. Scalar measurement invariance between adolescent subgroups was supported for the MHS: A [age: χ^2^(172) = 756.422, *p* < 0.001, CFI = 0.918, SRMR = 0.054, RMSEA = 0.055 (90% CI 0.051–0.059); sex: χ^2^(108) = 620.856, *p* < 0.001, CFI = 0.925, SRMR = 0.044, RMSEA = 0.053 (90% CI 0.049–0.057)]. Scalar measurement invariance across sex subgroups and partial scalar measurement invariance across age subgroups were supported for the MHS: S [age: χ^2^(16) = 16.440, *p* <0.001, CFI = 1, SRMR = 0.033, RMSEA = 0.005 (90% CI 0.000–0.028); sex: χ^2^(16) = 27.680, *p* <0.001, CFI = 0.982, SRMR = 0.037, RMSEA = 0.033 (90% CI 0.018–0.047)]. Scalar measurement invariance between age subgroups was not supported for Items 2 (suicidal thoughts) and 3 (past attempts), and intercepts of Item 2 (elementary) and Item 4 (high school) are freely estimated based on the MI of each item, and the partial scalar measurement invariance of MHS: S between age groups was met.

### 3.4 Normative data displayed by percentile ranks for the total scores of MHS tools

Supplementary Tables S3–S5 present normative data for the MHS: D (Supplementary Table S3), MHS: A (Supplementary Table S4), and MHS: S (Supplementary Table S5) for different age and sex groups. The percentiles in the tables can be used to determine where an individual’s MHS score falls in comparison to the normative population as a whole and to the normative population of adolescents in the individual’s sex and age group. The percentile rank tends to decrease with age and among females. For example, for the MHS: D cutoff of 17, the overall norm percentile is 93.2, but it is 97.7 for elementary school males and 88.8 for high school females (Supplementary Table S4).

### 3.5 Correlations with other clinical symptoms and COVID-19-related stress

Higher MHS: D, MHS: A, and MHS: S scores were significantly associated with self-harm, somatization, perceived stress, and COVID-19-related stress indicators (all *p* < 0.005; Table 3). Pearson’s correlation coefficients showed that the MHS: D scores are highly correlated with self-harm (*r* = 0.357), somatization (*r* = 0.552), and perceived stress (*r* = 0.627). The MHS: A scores are highly correlated with self-harm (*r* = 0.346), somatization (*r* = 0.599), and perceived stress (*r* = 0.620). The MHS: S exhibited moderate to high correlations with self-harm (*r* = 0.385), somatization (*r* = 0.429), and perceived stress (*r* = 0.443). Strong correlations between COVID-19-related stress and the MHS: D (*r* = 0.607), MHS: A (*r* = 0.636), and MHS: S (*r* = 0.478) were observed.

**Table 3:** Correlations between MHSs and clinical indicators.

Tools	Self-harm	Somatization	Perceived stress	COVID-19 distress
MHS: D	0.357	0.552	0.627	0.607
MHS: A	0.346	0.599	0.62	0.636
MHS: S	0.385	0.429	0.443	0.478

## 4 Discussion

For the South Korean youth population, this is the first standardization and validation study of mental health scales, conducted with a large representative sample of adolescents (*N* = 6,689). This study provided the psychometric properties and normative data of three MHS tools developed in South Korea: MHS: D, MHS: A, and MHS: S.

### 4.1 Psychometric properties and normative data of MHS tools for South Korean adolescents

The three tools had good-to-excellent reliability when administered to an adolescent normative sample (MHS: D α = 0.92, MHS: A α = 0.93, and MHS: S α = 0.86) and acceptable goodness-of-fit values in factor analyses. We also confirmed that all three instruments had a single-factor structure, as in the adult population, and that they measured depression, anxiety, and suicide risk equivalently in adult and adolescent populations. The finding that MHS tools in adult and adolescent populations have the same psychometric properties and measurement invariance suggests that cutoff points for depressive and anxiety disorders and suicidality derived from studies of structured diagnostic-based MHS tools for adults could be applicable to adolescents.

This study also provided normative data for depression, anxiety, and suicide risk instruments in a sample of 6,689 South Korean adolescents. Normative data with percentile ranks according to total scores would provide clinicians with more information about an individual’s mental health. The reference norms would also provide an easier and more informative interpretation for adolescents who want to know their mental health status accurately.

### 4.2 Measurement invariance and developmental difference between demographic subgroups

Measurement invariance comparison of adolescent subgroup scores found that the adolescent screening tools equivalently measured depression, anxiety, and suicide risk regardless of one’s age and sex, and this indicates that the scores could be generalized across different demographic characteristics of the screening population.

The measurement invariance tests also indicated that some single items should be used with caution when interpreting psychological symptoms, considering differences in adolescents’ developmental characteristics. For Item 12 of the MHS: D (insomnia), which did not meet full scalar measurement invariance between adolescents and adults, this likely reflects that insomnia is a common symptom of depression in adolescents (Roberts et al., 1995). However, there is no significant association between insomnia and the onset of major depressive disorder in adolescents (Rice et al., 2019). Therefore, rather than interpreting insomnia as having a specific effect on depression in adolescents, this finding may reflect a developmental characteristic of adolescence, in which adolescents report sleep difficulties more frequently than adults (de Zambotti et al., 2018); and a cultural characteristic, in which Asian adolescents sleep less than other cultures (Olds et al., 2010). Psychomotor abnormalities and appetite changes were non-full-scalar measurement invariant across adolescent age groups. The intercept difference in the psychomotor abnormalities item may replicate previous findings that older adolescents with depression are more likely to experience psychomotor symptoms (Baji et al., 2009), which appear to be strongly associated with psychomotor changes owing to neurodevelopmental changes during adolescence (Cioni and Sgandurra, 2013). Appetite changes might be influenced by hormonal changes that occur during the rapid growth process of adolescence and cultural factors, such as the unique adolescent culture of body dissatisfaction (Maxwell and Cole, 2009) that is notable in South Korea (Kim, 2018). In the case of suicide ideation and attempt items, this appears to reflect the tendency for the rate of suicidal ideation to spike after 14 years of age (Nock et al., 2013). These items are a comprehensive measure of suicide attempts with no time limit; thus, older individuals may have accumulated suicide attempts and consequently scored higher on the relevant items. Therefore, caution should be exercised when using specific item scores in isolation to interpret mental health problems in adolescents (MHS: D Items 3, 10/11, and 12; MHS: S Items: 2 and 4), considering the developmental and cultural characteristics of South Korean adolescents.

Notably, the interpretation of the sum and mean differences in scores for each group is not compromised even if full scalar invariance is violated for a few items (Alvarez et al., 2016; Steenkamp and Baumgartner, 1998). Although the age-specific youth psychometric and general characteristics explained above made some screening tools achieve only partial measurement invariance, all three MHS instruments are equivalent measures of mental health problems in adolescents of different ages and sexes.

Normative data indicate that girls tend to have lower percentile ranks than boys, and older age groups tend to have lower percentile ranks than younger age groups for the same total score. This means that a higher proportion of girls compared to boys, and older age groups compared to younger ones, are at risk for depression, anxiety, and suicidal risk. This result aligns with former findings that older age and female sex are associated with greater vulnerability to internalized psychological problems and higher suicidality (Copeland et al., 2014; Lee, 2012; Lu, 2019; Nock et al., 2013; Oh and Seon, 2013). Therefore, the results of the MHS should be interpreted in relation to the percentile rank for each age and sex. For example, an MHS: D score of 13 for male elementary school seniors may be considered a level of depression that does not reach the clinical cutoff, but they are experiencing severe depression (top 5%) compared to other male senior elementary school peers.

In addition, even if the percentile rank of scores obtained is low compared to the overall norm and identifies a relatively common mental health problem, it would be misleading to interpret that the severity of mental health problems decreases. For example, MHS: S scores of 3 or higher are relatively common among female high-school-level adolescents: about 1 in 10 (89.5% in sum score 3). However, given that the MHS tools’ measurement consistency has been met in adults and adolescents, the score results should be still interpreted that she has a high suicide risk, as the score indicates that she is in the high-risk group according to the MHS: S cutoff.

The findings ensure that developmental and psychosocial factors in adolescents significantly influence mental health score differences, but not to the extent that they distort measurement invariance between demographic subgroups. Various factors appear to contribute to psychopathology in adolescents, including biological vulnerabilities; gender role differences; the effects of physical, cognitive, and emotional development during puberty; and academic performance and peer relationships (Franić et al., 2010; Kashani and Orvaschel, 1990; Makri-Botsari, 2005). Therefore, aligning with the results of measurement invariance and norm difference between age subgroups, this study emphasizes that though MHS tools could be used universally in South Korean adolescents, score differences between age and sex groups should be interpreted considering understanding the unique developmental and cultural context of an adolescent.

### 4.3 Correlation between MHS tools and negative mental health outcomes

The MHS: D, MHS: A, and MHS: S were all highly correlated with scores on instruments indicating mental health risk, suggesting that all three instruments had adequate construct validity. In previous studies, poor adolescent mental health, such as depression, anxiety, and suicidality, were highly associated with maladaptive responses, such as somatization and self-harm (Hawton et al., 2013; Lipowski, 1988; Löwe et al., 2008b; Moran et al., 2012) and perceived stress levels (Chen and Kuo, 2020; Lee, 2012). All three screening tools were highly positively correlated with somatization, self-harm, and perceived stress, which confirms that they are accurate measures of mental health issues. In addition to confirming the construct validity of the MHS instrument, we also confirmed that adolescent depression and anxiety are risk factors for high somatization and self-harm. We ensured that untreated common mental health concerns could contribute to worsening the quality of life and even the overall health and safety of adolescents.

The strong association between COVID-19-related stress and depression, anxiety, and suicide risk found in this study aligns with studies showing that the pandemic and social distancing measures significantly impacted public mental health. Prolonged social distancing in South Korea owing to the pandemic introduced new stressors, such as technical difficulties, decreased concentration, and lack of motivation, which adversely impacted academic performance and mental health (Adnan and Anwar, 2020; Branje and Morris, 2021; Huang and Ougrin, 2021; Marciano et al., 2022; Stewart et al., 2022). These conditions might also have led to an increase in depression, anxiety, and suicide risk among adolescents observed in this study, and vice versa, especially when one who has depression and anxiety symptoms is prone to experience high stress owing to COVID-19. Because evidence is mixed as to whether COVID-19 negatively affected the prevalence of depression, anxiety, and suicidal ideation in the South Korean adolescent population (Jo et al., 2023; Kim et al., 2023), this issue needs further exploration.

Although the global prevalence of depression, anxiety, and suicide risk increases as adolescents age, there is a particular problem that comes with increasing school levels in South Korea. For Korean adolescents, admission to top colleges is overly emphasized as a prerequisite for a successful future. Students must earn high scores on the *Suneung* (College Scholastic Ability Test) and maintain good *Naeshin* (GPA in high school) to be accepted. In general, they are advised to spend their entire day studying for the *Suneung* and *Naeshin* subjects, and this intense preparation begins in earnest in high school around age 16 (making things worse, most adolescents begin preparing for this critical stage in middle school and even earlier in elementary school). During this time, they experience extreme pressure to be perfect academically. However, rather than providing them with coping strategies to handle this harsh situation, their parents and the school environment implicitly or explicitly force them to be more competitive in order to achieve “high.” Surely, this unique competitive circumstances have worsened overall mental health problems of Korean adolescents (Kim, 2024; Park and Chung, 2014), compared to one from any other cultural backgrounds. Even for youth who have dropped out of school for academic, health, or other reasons, having inherently internalized the label of having dropped out midway in the “normal” path to “success,” lack of occupational support and social stigma against out-of-school youth could make them vulnerable to mental health threats (Chae and Bae, 2024). All groups of South Korean adolescents have been facing special psychosocial challenges, leading to chronic mental health issues, resulting in an increased risk of suicide.

To address these, providing continuous mental health monitoring with screening tools is essential, which could help subsequent diagnosis and preventive measures and interventions (Daniunaite et al., 2021; Loy et al., 2024). In South Korea, adolescents are regularly screened for emotional and behavioral problems at school every three years (Student Emotional and Behavioral Screening Test). However, these screenings are limited to identifying general mental health problems and are not intended to screen for specific mental health problems such as general anxiety disorder, depressive disorders, and suicide risk, resulting in limited prevention and treatment for specific symptoms. Further, this regular screening excludes out-of-school adolescents. The use of validated, evidence-based screening tools in community and in-and-out-of-school settings for youth will help individuals and professionals accurately assess their own mental health and suicide risk, allowing for more appropriate and personalized prevention and intervention. The use of validated MHS tools in conjunction with the sex-and age-specific norms will provide more accurate information about the severity of a client’s mental health problems.

### 4.4 Limitations and future research

This study had some limitations. First, it was conducted with South Korean adolescents, which may limit its generalizability to other cultures. Future studies should examine the reliability and validity of these instruments among adolescents from different cultural backgrounds, and cross-cultural measurement consistency by collecting measurement data with MHS tools from different cultural backgrounds and conducting measurement invariance tests with the normative sample in this study (i.e., Stevanovic et al., 2017), to gain universality of these tools. Second, diagnosis by a clinician through a structured interview was not included. Therefore, the MHS cut-off points for adolescents could remain provisional, as the associations between total scores and actual diagnoses have not yet been analyzed. However, validation studies of the MHS: D, MHS: A, and MHS: S in adults included structured interviews to demonstrate the diagnostic validity of the screening tools (Kim et al., 2021; Park et al., 2022; Yoon et al., 2020), succeeding to set the exact cut-off points of depression, anxiety and suicide risk for adults, and this study confirmed that each MHS tool could measure consistently in both adult and adolescent populations and that the adult cut-off points be applied to adolescents the same, at least tentatively. Future studies should include structured interviews with adolescents to rigorously establish cutoff points for major depressive disorder, generalized anxiety disorder, and suicidality.

In addition, although many out-of-school youth were sampled in this survey, the study focus was on standardizing and validating the MHS in the South Korean adolescent population, which prevented further exploration of mental health and suicide risk characteristics in out-of-school populations. Further, although the year 2020–2022 was a period of sustained negative effects of social distancing owing to COVID-19, analysis of the long-term impact of the pandemic on participants’ mental health is limited as we employed a cross-sectional design. During a period of significant pandemic-related restrictions, the survey was conducted and confirmed strong associations between pandemic-related stress and depression, anxiety, and suicide risk. However, since this is a cross-sectional study that only examines a specific point in time, interpretations of causal relationships between variables (e.g., that worsening depression levels in adolescents may have been caused by increased pandemic-related distress) should be limited. A longitudinal study could be conducted to examine the causal relationship between COVID-induced distress and mental health. Finally, few attempts in the survey were made to identify or control factors that could influence biased and subjective responses among adolescents, such as underreporting or overreporting due to peer pressure, or differences in literacy of adolescents. Future studies aiming to validate screening tools for adolescent population should incorporate research designs that account for or control response bias.

The strength of this study is that it used data from over 6,000 adolescents nationwide to confirm the psychometric properties of screening tools. This study analyzed all adolescent age groups, not just those in specific school grades, including out-of-school adolescents. Using the adolescent sample with out-of-school participants enhanced the generalizability of the results for South Korean adolescents with different educational backgrounds because these youths are more vulnerable to emotional, adjustment, and environmental challenges than in-school adolescents, and this might impact the overall mental health status of adolescents. Further, measurement invariance across age groups confirmed that the MHS tools are valid measures of mental health for all adolescents in South Korea. In addition, survey data were collected online, which can be used as a basis for incorporating the tools into applications and online platforms targeting adolescents. Therefore, this study provides evidence that these screening tools can be used to measure depression, anxiety, and suicidality in adolescents, and explore the detrimental effect of mental health concerns on the overall quality of life and safety of South Korean adolescents.

### 4.5 Conclusion

The MHS: D, MHS: A, and MHS: S demonstrated good-to-excellent reliability and validity in a sample of South Korean adolescents. Each instrument also demonstrated a unidimensional construct and adequate measurement invariance across sex and age subgroups. This study also provided youth normative data for the MHS, which makes the MHS easier to interpret. Adolescent depression and anxiety are correlated with worsening mental health and increased suicide risk, and the detrimental effects of COVID-19 on adolescents’ health problems were observed. Unresolved psychosocial problems in South Korea are a major contributor to the high suicide risk among adolescents. However, adolescent mental health problems are preventable public health issues. These screening tools with normative references are highly useful for clinicians in community settings and on web-based platforms for identifying ongoing depression, anxiety, and suicidality in adolescents.

## Data Availability

The data analyzed in this study is subject to the following licenses/restrictions: The datasets used and/or analyzed during the current study are available from the corresponding author on reasonable request. Requests to access these datasets should be directed to david0177@korea.ac.kr.
