# Correction: Screening mental health risks for adolescents in South Korea

**DOI:** 10.3389/fpsyg.2025.1692835

**Published:** 2025-09-09

**Authors:** Frontiers Production Office

**Affiliations:** Frontiers Media SA, Lausanne, Switzerland

**Keywords:** suicide risk, depression, anxiety, adolescence, validation, screening tool, public mental health, COVID-19

The affiliation for reviewer Seyma Erdem Torun was incorrectly published as “Yuksek Ihtisas Training and Research Hospital, Türkiye”. The corrected affiliation is shown below:

“Ankara Bilkent City Hospital, Türkiye”.

The original version of this article has been updated.

