# Supplementary material for: Screening mental health risks for adolescents in South Korea
Source: Front Psychol. 2025 Jul 16;16:1589136. doi: 10.3389/fpsyg.2025.1589136 (PMC12308555; doi:10.3389/fpsyg.2025.1589136)
Supplement: Supplementary file 1 [file Table_1.DOCX]

Supplementary Material

**Table S1.** Measurement invariance between adult/adolescent samples

**Table S2.** Measurement invariance in adolescent samples

**Table S3.** MHS:D Normative data across demographic subgroups

**Table S4.** MHS:A Normative data across demographic subgroups

**Table S5.** MHS:S Normative data across demographic subgroups

**Table S1.** Measurement invariance between adult/adolescent samples

| **Model** | **Model Fit statistics** | | | | **Measurement invariance test statistics** | | | |
| --- | --- | --- | --- | --- | --- | --- | --- | --- |
|  | $\boldsymbol{x}^{\boldsymbol{2}}$**(df)** | **CFI** | **SRMR** | **RMSEA(90% CI)** | **Δ CFI** | **Δ SRMR** | **ΔRMSEA** | **Decision** |
| *Adult/adolescent* |  |  |  |  |  |  |  |  |
| **MHS:D** |  |  |  |  |  |  |  |  |
| 1. Configural | 923.718(88) | 0.930 | 0.038 | 0.070(0.066~0.074) |  |  |  |  |
| 2. Metric(vs. 1) | 950.811(98) | 0.929 | 0.039 | 0.067(0.063~0.071) | -0.001 | 0.001 | -0.003 | ACCEPT |
| 3. Scalar(vs. 2) | 1091.096(108) | 0.918 | 0.042 | 0.069(0.065~0.072) | -0.011 | 0.003 | 0.003 | REJECT |
| 3a. partial Scalar(vs. 2)* | 1016.468(105) | 0.920 | 0.041 | 0.068(0.064~0.072) | -0.009 | 0.002 | 0.001 | ACCEPT |
| **MHS:A** |  |  |  |  |  |  |  |  |
| 1. Configural | 896.510(88) | 0.930 | 0.041 | 0.069(0.065~0.073) |  |  |  |  |
| 2. Metric(vs. 1) | 986.762(98) | 0.923 | 0.048 | 0.068(0.065~0.072) | -0.007 | 0.007 | -0.001 | ACCEPT |
| 3. Scalar(vs. 2) | 1094.141(108) | 0.914 | 0.049 | 0.069(0.065~0.072) | -0.009 | 0.001 | 0.001 | ACCEPT |
| **MHS:S** |  |  |  |  |  |  |  |  |
| 1. Configural | 22.427(4) | 0.989 | 0.018 | 0.049(0.030~0.069) |  |  |  |  |
| 2. Metric(vs. 1) | 29.197(7) | 0.986 | 0.028 | 0.040(0.026~0.056) | -0.003 | 0.01 | 0.009 | ACCEPT |
| 3. Scalar(vs. 2) | 52.151(10) | 0.974 | 0.032 | 0.047(0.035~0.060) | -0.012 | 0.004 | 0.007 | REJECT |
| 3a. Partial Scalar(vs.2)** | 16.440(16) | 0.979 | 0.031 | 0.044(0.032~0.058) | -0.007 | 0.003 | 0.004 | ACCEPT |
| ** Intercept of item12 is freely estimated. ** Intercept of item2 is freely estimated*  *CFI = comparative fit index; CI = confidence interval; df = degrees of freedom; RMSEA = root mean standard error of approximation; SRMR = square root mean residual*. *Δ = index difference between the model and the one in the parenthesis* | | | | | | | | |

**Table S2.** Measurement invariance in adolescent samples

| **Model** | **Model Fit statistics** | | | | **Measurement invariance test statistics** | | | |
| --- | --- | --- | --- | --- | --- | --- | --- | --- |
|  | $\boldsymbol{x}^{\boldsymbol{2}}$**(df)** | **CFI** | **SRMR** | **RMSEA(90% CI)** | **Δ CFI** | **Δ SRMR** | **ΔRMSEA** | **Decision** |
| *Age (Elementary (4-6) vs. Middle vs. High)* |  |  |  |  |  |  |  |  |
| **MHS:D** |  |  |  |  |  |  |  |  |
| 1. Configural | 655.347(132) | 0.932 | 0.04 | 0.060(0.055~0.064) |  |  |  |  |
| 2. Metric(vs. 1) | 686.000(152) | 0.931 | 0.049 | 0.056(0.052~0.060) | -0.001 | 0.009 | -0.004 | ACCEPT |
| 3. Scalar(vs. 2) | 784.991(172) | 0.92 | 0.051 | 0.057(0.053~0.061) | -0.011 | 0.002 | 0.001 | REJECT |
| 3a. partial Scalar(vs. 2)* | 745.029(168) | 0.925 | 0.049 | 0.056(0.051~0.060) | -0.006 | 0 | 0 | ACCEPT |
| **MHS:A** |  |  |  |  |  |  |  |  |
| 1. Configural | 663.757(132) | 0.925 | 0.044 | 0.060(0.056~0.065) |  |  |  |  |
| 2. Metric(vs. 1) | 690.491(152) | 0.924 | 0.054 | 0.056(0.052~0.061) | -0.001 | 0.01 | -0.004 | ACCEPT |
| 3. Scalar(vs. 2) | 756.422(172) | 0.918 | 0.054 | 0.055(0.051~0.059) | -0.006 | 0 | -0.001 | ACCEPT |
| **MHS:S** |  |  |  |  |  |  |  |  |
| 1. Configural | 11.215(6) | 0.994 | 0.019 | 0.028(0.000~0.053) |  |  |  |  |
| 2. Metric(vs. 1) | 12.124(12) | 1 | 0.032 | 0.003(0.000~0.031) | 0.006 | 0.013 | 0.002 | ACCEPT |
| 3. Scalar(vs. 2) | 26.287(18) | 0.991 | 0.039 | 0.020(0.000~0.036) | -0.009 | 0.02 | 0.017 | REJECT |
| 3a. Partial Scalar(vs.2)** | 16.440(16) | 1 | 0.033 | 0.005(0.000~0.028) | 0 | 0.001 | 0.002 | ACCEPT |
| *Sex (Girls vs. Boys)* |  |  |  |  |  |  |  |  |
| **MHS:D** |  |  |  |  |  |  |  |  |
| 1. Configural | 684.639(88) | 0.926 | 0.042 | 0.064(0.059~0.068) |  |  |  |  |
| 2. Metric(vs. 1) | 716.379(98) | 0.923 | 0.049 | 0.061(0.057~0.066) | -0.003 | 0.007 | -0.003 | ACCEPT |
| 3. Scalar(vs. 2) | 778.317(108) | 0.917 | 0.049 | 0.061(0.057~0.065) | -0.006 | 0 | 0 | ACCEPT |
| **MHS:A** |  |  |  |  |  |  |  |  |
| 1. Configural | 576.842(88) | 0.93 | 0.042 | 0.058(0.053~0.062) |  |  |  |  |
| 2. Metric(vs. 1) | 578.433(98) | 0.932 | 0.043 | 0.054(0.050~0.058) | 0.002 | 0.001 | -0.004 | ACCEPT |
| 3. Scalar(vs. 2) | 620.856(108) | 0.927 | 0.044 | 0.053(0.049~0.057) | -0.005 | 0.001 | -0.001 | ACCEPT |
| **MHS:S** |  |  |  |  |  |  |  |  |
| 1. Configural | 15.527(4) | 0.988 | 0.018 | 0.042(0.021~0.064) |  |  |  |  |
| 2. Metric(vs. 1) | 18.758(7) | 0.988 | 0.036 | 0.032(0.015~0.049) | 0 | 0.018 | -0.01 | ACCEPT |
| 3. Scalar(vs. 2) | 27.680(10) | 0.982 | 0.037 | 0.033(0.018~0.047) | -0.006 | 0.001 | 0.001 | ACCEPT |
| *Note. * Intercepts of item10/11(all groups), item3(elementary) are freely estimated. ** Intercepts of item2(elementary) and item4(high) are freely estimated.*  *CFI = comparative fit index; CI = confidence interval; df = degrees of freedom; RMSEA = root mean standard error of approximation; SRMR = square root mean residual*. *Δ = index difference between the model and the one in the parenthesis* | | | | | | | | |

**Table S3.** MHS:D Normative data across demographic subgroups

| **MHS:D** | | | | | | | | | |
| --- | --- | --- | --- | --- | --- | --- | --- | --- | --- |
| **Sum Score** | **Percentile** | | | | | | | | |
|  | **Total** | **Boys** | | | | **Girls** | | | |
|  | **n=6689** | **Age 10-12** | **Age 13-15** | **Age 16-18** | **Total** | **Age 10-12** | **Age 13-15** | **Age 16-18** | **Total** |
|  |  | **n=1004** | **n=1080** | **n=1020** | **n=3104** | **n=1050** | **n=958** | **n=1577** | **n=3585** |
| **0** | 31.1 | 30.0 | 37.6 | 35.7 | 34.5 | 29.6 | 29.7 | 26.0 | 28.1 |
| **1** | 44.6 | 45.7 | 50.3 | 46.4 | 47.5 | 48.0 | 44.5 | 36.7 | 42.1 |
| **2** | 54.6 | 57.0 | 60.1 | 54.6 | 57.3 | 60.6 | 54.8 | 45.3 | 52.3 |
| **3** | 61.6 | 64.7 | 66.4 | 62.2 | 64.5 | 69.1 | 61.0 | 51.2 | 59.1 |
| **4** | 67.4 | 72.1 | 72.4 | 67.3 | 70.6 | 74.9 | 65.3 | 57.5 | 64.7 |
| **5** | 72.2 | 77.0 | 76.3 | 71.8 | 75.0 | 79.6 | 70.8 | 62.5 | 69.7 |
| **6** | 75.9 | 81.1 | 78.9 | 75.5 | 78.5 | 82.8 | 74.3 | 67.1 | 73.6 |
| **7** | 79.1 | 85.3 | 81.6 | 78.1 | 81.6 | 85.3 | 77.8 | 71.0 | 77.0 |
| **8** | 81.7 | 87.6 | 84.2 | 81.4 | 84.4 | 87.0 | 80.2 | 73.7 | 79.3 |
| **(Mild)9** | 83.6 | 89.8 | 85.8 | 83.3 | 86.3 | 88.4 | 82.0 | 76.2 | 81.3 |
| **10** | 85.1 | 91.0 | 87.3 | 84.9 | 87.7 | 89.5 | 83.5 | 78.1 | 82.9 |
| **11** | 86.8 | 92.4 | 88.8 | 87.0 | 89.4 | 91.0 | 84.2 | 80.5 | 84.5 |
| **12** | 88.3 | 93.9 | 89.8 | 88.5 | 90.7 | 92.5 | 85.6 | 82.5 | 86.2 |
| **(Moderate) 13** | 89.8 | 95.1 | 92.4 | 89.4 | 92.3 | 93.1 | 86.7 | 84.3 | 87.6 |
| **14** | 90.8 | 95.8 | 93.9 | 90.4 | 93.4 | 93.7 | 88.1 | 85.6 | 88.6 |
| **15** | 91.8 | 96.8 | 94.6 | 91.1 | 94.2 | 94.0 | 89.2 | 87.1 | 89.7 |
| **16** | 92.4 | 97.2 | 95.4 | 91.6 | 94.7 | 94.5 | 90.1 | 88.0 | 90.4 |
| **(Cut-off) 17** | 93.2 | 97.7 | 96.1 | 92.5 | 95.5 | 95.0 | 91.0 | 88.8 | 91.2 |
| **18** | 93.6 | 98.0 | 96.3 | 92.9 | 95.7 | 95.3 | 91.8 | 89.5 | 91.8 |
| **19** | 94.2 | 98.1 | 97.1 | 93.7 | 96.3 | 95.8 | 92.5 | 90.1 | 92.4 |
| **20** | 94.9 | 98.4 | 97.5 | 94.3 | 96.7 | 96.7 | 93.0 | 91.2 | 93.3 |
| **(Severe) 21** | 95.3 | 98.6 | 97.6 | 94.9 | 97.0 | 97.0 | 93.4 | 91.7 | 93.7 |
| **22** | 95.9 | 98.8 | 98.2 | 95.4 | 97.5 | 97.2 | 94.5 | 92.8 | 94.5 |
| **23** | 96.4 | 98.9 | 98.6 | 96.5 | 98.0 | 97.5 | 95.0 | 93.2 | 95.0 |
| **24** | 96.6 | 98.9 | 99.0 | 96.7 | 98.2 | 97.5 | 95.3 | 93.7 | 95.3 |
| **25** | 96.8 | 99.2 | 99.1 | 96.7 | 98.3 | 97.7 | 95.5 | 94.2 | 95.6 |
| **26** | 97.1 | 99.4 | 99.4 | 96.8 | 98.5 | 98.2 | 95.9 | 94.4 | 95.9 |
| **27** | 97.4 | 99.4 | 99.4 | 97.1 | 98.6 | 98.6 | 96.2 | 94.7 | 96.3 |
| **28** | 97.7 | 99.4 | 99.4 | 97.4 | 98.7 | 98.7 | 96.3 | 95.8 | 96.8 |
| **29** | 97.9 | 99.5 | 99.4 | 97.5 | 98.8 | 98.9 | 96.8 | 95.9 | 97.0 |
| **30** | 98.1 | 99.6 | 99.4 | 97.8 | 99.0 | 99.0 | 97.2 | 96.4 | 97.4 |
| **31** | 98.4 | 99.7 | 99.5 | 98.1 | 99.1 | 99.0 | 97.7 | 96.8 | 97.7 |
| **32** | 98.6 | 99.7 | 99.6 | 98.3 | 99.2 | 99.1 | 98.2 | 97.1 | 98.0 |
| **33** | 98.7 | 99.8 | 99.7 | 98.4 | 99.3 | 99.2 | 98.4 | 97.3 | 98.2 |
| **34** | 98.9 | 99.8 | 99.7 | 98.6 | 99.4 | 99.2 | 98.9 | 97.7 | 98.4 |
| **35** | 99.1 | > 99.9 | 99.7 | 98.7 | 99.5 | 99.6 | 98.9 | 98.0 | 98.7 |
| **36** | 99.2 | > 99.9 | 99.8 | 98.8 | 99.5 | 99.7 | 99.1 | 98.2 | 98.9 |
| **37** | 99.4 | > 99.9 | 99.8 | 99.1 | 99.6 | 99.8 | 99.5 | 98.5 | 99.2 |
| **38** | 99.5 | > 99.9 | 99.8 | 99.3 | 99.7 | 99.9 | 99.6 | 98.7 | 99.3 |
| **39** | 99.6 | > 99.9 | 99.8 | 99.3 | 99.7 | 99.9 | 99.7 | 99.0 | 99.4 |
| **40** | 99.7 | > 99.9 | 99.9 | 99.4 | 99.8 | 99.9 | 99.8 | 99.2 | 99.6 |
| **41** | 99.7 | > 99.9 | 99.9 | 99.4 | 99.8 | 99.9 | 99.9 | 99.2 | 99.6 |
| **42** | 99.7 | > 99.9 | 99.9 | 99.4 | 99.8 | 99.9 | 99.9 | 99.5 | 99.7 |
| **43** | 99.8 | > 99.9 | 99.9 | 99.6 | 99.8 | 99.9 | 99.9 | 99.6 | 99.8 |
| **44** | > 99.9 | > 99.9 | > 99.9 | > 99.9 | > 99.9 | > 99.9 | > 99.9 | > 99.9 | > 99.9 |
| **Mean(SD)** | 4.88(7.29) | 3.64(4.97) | 3.90(5.83) | 4.94(7.64) | 4.23(6.41) | 3.88(6.16) | 5.38(8.01) | 6.65(8.9) | 5.56(8.12) |

**Table S4.** MHS:A Normative data across demographic subgroups

| **MHS:A** | | | | | | | | | |
| --- | --- | --- | --- | --- | --- | --- | --- | --- | --- |
| **Sum Score** | **Percentile** | | | | | | | | |
|  | **Total** | **Boys** | | | | **Girls** | | | |
|  | **n=6689** | **Age 10-12** | **Age 13-15** | **Age 16-18** | **Total** | **Age 10-12** | **Age 13-15** | **Age 16-18** | **Total** |
|  |  | **n=1004** | **n=1080** | **n=1020** | **n=3104** | **n=1050** | **n=958** | **n=1577** | **n=3585** |
| **0** | 42.2 | 45.0 | 49.4 | 44.1 | 46.2 | 43.9 | 41.5 | 33.7 | 38.8 |
| **1** | 53.3 | 59.1 | 60.1 | 54.5 | 57.9 | 57.0 | 50.7 | 43.4 | 49.3 |
| **2** | 61.3 | 66.5 | 67.7 | 62.9 | 65.8 | 66.1 | 58.7 | 51.0 | 57.5 |
| **3** | 67.3 | 72.9 | 72.3 | 68.9 | 71.4 | 73.7 | 63.7 | 57.4 | 63.8 |
| **4** | 72.2 | 77.1 | 76.9 | 73.6 | 75.9 | 78.6 | 68.5 | 62.8 | 68.9 |
| **5** | 75.6 | 80.5 | 80.1 | 76.3 | 79.0 | 82.4 | 72.7 | 66.2 | 72.7 |
| **6** | 78.6 | 84.2 | 83.6 | 78.3 | 82.1 | 84.9 | 75.6 | 69.6 | 75.6 |
| **7** | 80.9 | 86.4 | 85.2 | 80.6 | 84.1 | 86.7 | 77.7 | 73.0 | 78.2 |
| **8** | 83.0 | 88.0 | 87.1 | 82.5 | 85.9 | 88.5 | 80.3 | 75.1 | 80.4 |
| **9** | 84.6 | 89.9 | 88.4 | 84.2 | 87.5 | 89.0 | 81.3 | 77.7 | 82.0 |
| **(Mild) 10** | 86.0 | 91.5 | 89.4 | 85.3 | 88.8 | 90.3 | 83.1 | 79.6 | 83.7 |
| **11** | 87.9 | 93.0 | 91.7 | 87.2 | 90.6 | 91.5 | 85.1 | 81.7 | 85.5 |
| **12** | 89.0 | 93.9 | 92.4 | 88.5 | 91.6 | 91.9 | 86.2 | 83.4 | 86.7 |
| **13** | 90.0 | 94.2 | 93.4 | 89.8 | 92.5 | 93.0 | 87.3 | 84.6 | 87.8 |
| **14** | 90.7 | 94.8 | 94.1 | 90.2 | 93.0 | 94.0 | 88.5 | 85.4 | 88.8 |
| **(Cutoff) 15** | 91.5 | 95.3 | 94.5 | 91.4 | 93.8 | 95.0 | 89.1 | 86.3 | 89.6 |
| **16** | 92.3 | 96.3 | 95.2 | 92.0 | 94.5 | 95.7 | 90.4 | 87.1 | 90.5 |
| **17** | 93.1 | 96.7 | 96.1 | 92.5 | 95.1 | 95.9 | 91.4 | 88.3 | 91.4 |
| **18** | 93.7 | 97.1 | 96.7 | 93.2 | 95.7 | 96.7 | 91.9 | 89.0 | 92.0 |
| **19** | 94.1 | 97.7 | 97.2 | 93.4 | 96.1 | 96.8 | 92.3 | 89.3 | 92.3 |
| **(Moderate) 20** | 94.6 | 98.0 | 97.5 | 94.0 | 96.5 | 97.2 | 92.7 | 90.2 | 92.9 |
| **21** | 95.0 | 98.2 | 97.9 | 94.6 | 96.9 | 97.4 | 93.1 | 90.8 | 93.3 |
| **22** | 95.7 | 98.5 | 98.3 | 95.4 | 97.4 | 97.8 | 94.1 | 92.0 | 94.1 |
| **23** | 96.2 | 99.0 | 98.6 | 96.0 | 97.9 | 97.8 | 94.7 | 92.9 | 94.8 |
| **24** | 96.7 | 99.1 | 98.8 | 96.6 | 98.2 | 98.2 | 95.2 | 93.7 | 95.4 |
| **25** | 97.1 | 99.3 | 99.0 | 97.2 | 98.5 | 98.3 | 95.7 | 94.4 | 95.9 |
| **26** | 97.5 | 99.3 | 99.1 | 97.5 | 98.6 | 98.6 | 96.1 | 95.4 | 96.5 |
| **27** | 97.8 | 99.3 | 99.3 | 97.5 | 98.7 | 98.9 | 96.7 | 95.9 | 97.0 |
| **28** | 98.0 | 99.4 | 99.4 | 97.6 | 98.8 | 99.0 | 97.2 | 96.1 | 97.2 |
| **29** | 98.3 | 99.6 | 99.4 | 98.1 | 99.1 | 99.2 | 97.4 | 96.8 | 97.7 |
| **(Severe) 30** | 98.6 | 99.7 | 99.5 | 98.4 | 99.2 | 99.4 | 97.9 | 97.1 | 98.0 |
| **31** | 98.7 | 99.7 | 99.6 | 98.5 | 99.3 | 99.6 | 98.2 | 97.3 | 98.2 |
| **32** | 98.8 | 99.7 | 99.6 | 98.5 | 99.3 | 99.6 | 98.4 | 97.5 | 98.4 |
| **33** | 99.1 | 99.9 | 99.8 | 98.8 | 99.5 | 99.6 | 98.7 | 98.2 | 98.8 |
| **34** | 99.2 | 99.9 | 99.8 | 99.0 | 99.6 | 99.6 | 99.0 | 98.5 | 98.9 |
| **35** | 99.3 | 99.9 | 99.8 | 99.2 | 99.6 | 99.7 | 99.1 | 98.7 | 99.1 |
| **36** | 99.4 | 99.9 | 99.9 | 99.2 | 99.7 | 99.7 | 99.2 | 98.9 | 99.2 |
| **37** | 99.5 | 99.9 | 99.9 | 99.3 | 99.7 | 99.7 | 99.3 | 99.1 | 99.3 |
| **38** | 99.6 | 99.9 | 99.9 | 99.4 | 99.7 | 99.7 | 99.4 | 99.2 | 99.4 |
| **39** | 99.6 | > 99.9 | 99.9 | 99.5 | 99.8 | 99.8 | 99.4 | 99.2 | 99.4 |
| **40** | 99.7 | > 99.9 | 99.9 | 99.5 | 99.8 | 99.9 | 99.4 | 99.4 | 99.5 |
| **41** | 99.7 | > 99.9 | 99.9 | 99.5 | 99.8 | > 99.9 | 99.4 | 99.5 | 99.6 |
| **42** | 99.8 | > 99.9 | 99.9 | 99.5 | 99.8 | > 99.9 | 99.7 | 99.6 | 99.7 |
| **43** | 99.9 | > 99.9 | 99.9 | 99.7 | 99.9 | > 99.9 | > 99.9 | 99.7 | 99.9 |
| **44** | > 99.9 | > 99.9 | > 99.9 | > 99.9 | > 99.9 | > 99.9 | > 99.9 | > 99.9 | > 99.9 |
| **Mean(SD)** | 4.35(7.23) | 3.11(5.25) | 3.24(5.66) | 4.38(7.51) | 3.57(6.24) | 3.30(5.83) | 5.04(8.07) | 6.15(8.81) | 5.02(7.93) |

**Table S5.** MHS:S Normative data across demographic subgroups

| **MHS:S** | | | | | | | | | |
| --- | --- | --- | --- | --- | --- | --- | --- | --- | --- |
| **Sum Score** | **Percentile** | | | | | | | | |
|  | **Total** | **Boys** | | | | **Girls** | | | |
|  | **n=6689** | **Age 10-12** | **Age 13-15** | **Age 16-18** | **Total** | **Age 10-12** | **Age 13-15** | **Age 16-18** | **Total** |
|  |  | **n=1004** | **n=1080** | **n=1020** | **n=3104** | **n=1050** | **n=958** | **n=1577** | **n=3585** |
| **0** | 81.0 | 86.5 | 85.9 | 83.9 | 85.4 | 80.5 | 79.5 | 73.4 | 77.1 |
| **(Low Risk) 1** | 87.9 | 92.3 | 91.9 | 89.4 | 91.2 | 88.0 | 86.6 | 82.1 | 85.0 |
| **2** | 92.1 | 95.2 | 94.5 | 92.8 | 94.2 | 93.3 | 90.8 | 87.8 | 90.2 |
| **(High Risk) 3** | 93.8 | 96.8 | 96.1 | 94.5 | 95.8 | 95.2 | 92.8 | 89.5 | 92.1 |
| **4** | 95.4 | 98.0 | 97.7 | 96.3 | 97.3 | 96.1 | 94.5 | 91.6 | 93.7 |
| **5** | 96.3 | 98.6 | 98.0 | 96.9 | 97.8 | 96.9 | 95.4 | 93.7 | 95.1 |
| **6** | 97.1 | 99.1 | 98.4 | 97.5 | 98.3 | 97.7 | 96.6 | 94.7 | 96.1 |
| **7** | 97.6 | 99.4 | 98.7 | 97.7 | 98.5 | 98.1 | 97.3 | 95.6 | 96.8 |
| **8** | 98.2 | 99.7 | 99.4 | 98.1 | 99.0 | 98.4 | 98.0 | 96.5 | 97.5 |
| **9** | 98.6 | 99.7 | 99.6 | 98.3 | 99.2 | 98.9 | 98.6 | 97.0 | 98.0 |
| **10** | 98.9 | 99.7 | 99.7 | 98.7 | 99.4 | 99.0 | 98.9 | 97.8 | 98.4 |
| **11** | 99.1 | 99.9 | 99.7 | 98.8 | 99.5 | 99.1 | 99.2 | 98.2 | 98.7 |
| **12** | 99.3 | 99.9 | 99.7 | 99.2 | 99.6 | 99.5 | 99.3 | 98.7 | 99.1 |
| **13** | 99.5 | 99.9 | 99.7 | 99.3 | 99.6 | 99.7 | 99.6 | 99.2 | 99.4 |
| **14** | 99.7 | 99.9 | 99.7 | 99.4 | 99.7 | 99.8 | 99.7 | 99.5 | 99.6 |
| **15** | 99.7 | 99.9 | 99.7 | 99.5 | 99.7 | 99.9 | 99.9 | 99.6 | 99.8 |
| **16** | > 99.9 | > 99.9 | > 99.9 | > 99.9 | > 99.9 | > 99.9 | > 99.9 | > 99.9 | > 99.9 |
| **Mean(SD)** | 0.66(2.0) | 0.36(1.28) | 0.41(1.48) | 0.60(2.02) | 0.46(1.63) | 0.60(1.83) | 0.73(2.08) | 1.05(2.59) | 0.83(2.26) |
